# PDGrad: Guiding Diffusion Model for Reference-Based Blind Face Restoration with Pivot Direction Gradient Guidance

**DOI:** 10.3390/s24227112

**Published:** 2024-11-05

**Authors:** Geon Min, Tae Bok Lee, Yong Seok Heo

**Affiliations:** 1 Department of Artificial Intelligence, Ajou University, Suwon 16499, Republic of Korea; aming20@ajou.ac.kr (G.M.); dolphin0104@ajou.ac.kr (T.B.L.); 2 Department of Electrical and Computer Engineering, Ajou University, Suwon 16499, Republic of Korea

**Keywords:** reference-based blind face restoration, classifier guidance diffusion model, conflicting gradients

## Abstract

Reference-based blind face restoration (RefBFR) has gained considerable attention because it utilizes additional reference images to restore facial images in situations where the degradation is caused by unknown factors, making it particularly useful in real-world applications. Recently, guided diffusion models have demonstrated exceptional performance in this task without requiring training. They achieve this by integrating gradients of the losses where each loss reflects the different desired properties of the additional external images. However, these approaches fail to consider potential conflicts between gradients of multiple losses, which can lead to sub-optimal results. To address this issue, we introduce Pivot Direction Gradient guidance (PDGrad), a novel gradient adjustment method for RefBFR within a guided diffusion framework. To this end, we first define the loss function based on both low-level and high-level features. For loss at each feature level, both the coarsely restored image and the reference image are fully integrated. In cases of conflicting gradients, a pivot gradient is established for each level and other gradients are aligned to it, ensuring that the strengths of both images are maximized. Additionally, if the magnitude of the adjusted gradient exceeds that of the pivot gradient, it is adaptively scaled according to the ratio between the two, placing greater emphasis on the pivot. Extensive experimental results on the CelebRef-HQ dataset show that the proposed PDGrad significantly outperforms competitive approaches both quantitatively and qualitatively.

## 1. Introduction

Blind face restoration (BFR) aims to restore a high-quality (HQ) face image from a low-quality (LQ) image that has been degraded by unknown and complex factors, such as downsampling, blur, noise, and compression artifacts. BFR is a highly ill-posed problem because the unknown degradation makes it difficult to determine a single solution for a given LQ image, leading to multiple possible outcomes. Since facial images are sensitive to even subtle differences, having detailed information is essential for accurate restoration. By utilizing high-quality (HQ) reference images of the same individual, it becomes possible to achieve a high quality of image that is difficult to attain with BFR methods that do not use reference images. In this context, the reference-based blind face restoration (RefBFR) method has gained significant attention for its unique ability to leverage additional reference images to improve accuracy for practical scenarios. As a result, it can be applied for various applications, including face recognition [1,2], face detection [3,4,5] and age estimation [6,7].

Recently, several RefBFR studies [8,9,10,11,12,13] have been proposed based on deep learning [14]. Among these methods, PGDiff [13] has demonstrated outstanding performance in RefBFR using a training-free guided diffusion model [15]. It provides guidance to an unconditional diffusion model pre-trained for face image generation by incorporating the gradients of the losses during the reverse diffusion process. Their loss function is structured as a combination of multiple distances, each representing a specific desired attribute of the additional images. These include the coarsely restored image, generated using an external restorer such as CodeFormer [16], and the reference image, processed through the ArcFace network [1]. However, the guidance technique of PGDiff [13] may not be the optimal solution for RefBFR. This limitation arises from their gradients, which focus on using low-level information solely from the coarsely restored image, while relying on high-level information exclusively from the reference image. As a result, this approach fails to capture the crucial low-level details from the reference image and the high-level features from the coarsely restored image, leading to sub-optimal results. Moreover, the guidance derived from merely summing the gradients of multiple loss functions often results in sub-optimal results, as these gradients may be incompatible, causing conflicts.

**Figure 1:**
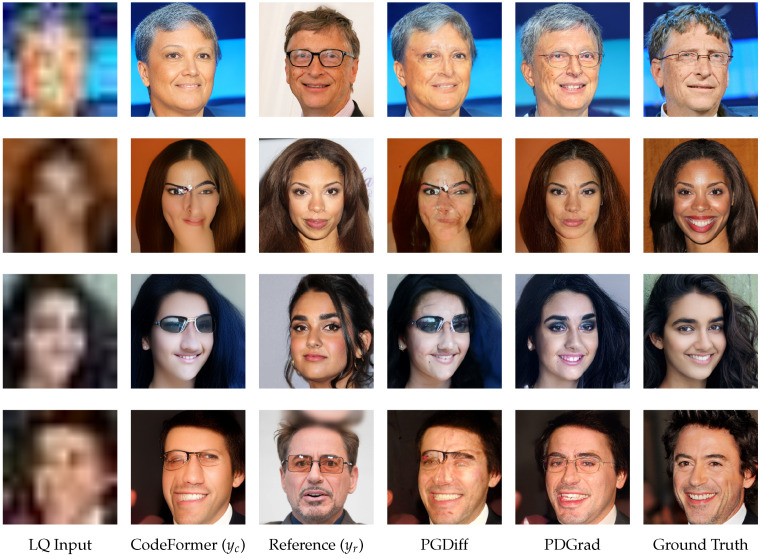
Example of restoration results for RefBFR. To obtain a coarsely restored image $y_{c}$ from the LQ input image, CodeFormer [16] is used as a restorer. Unlike PGDiff [13], which is significantly affected by the quality of $y_{c}$, the proposed PDGrad can generate images that mitigate this drawback.

To address this problem, we propose a novel gradient adjustment method for RefBFR called Pivot Direction Gradient guidance (PDGrad) within a guided diffusion framework. Inspired by PCGrad [17], the essence of our method is to reduce gradient interference by directly modifying the conflicting gradients of the loss. To this end, we first define the loss function based on both low-level and high-level features. Similar to PGDiff [13], we utilize external information such as the coarsely restored image $y_{c}$ which is obtained using the pre-trained restoration method such as CodeFormer [16] and the reference $y_{r}$. However, unlike PGDiff, we utilize both $y_{c}$ and $y_{r}$ to compute the loss at each level. This is because these two images capture complementary characteristics of face images. Figure 1 illustrates the complementary properties of $y_{c}$ and $y_{r}$. Generally, $y_{c}$ is aligned well with the LQ input and making it easy to compare with the prediction for low-level information such as edge, color and shape. However, certain areas of $y_{c}$ are not restored effectively. In contrast, $y_{r}$ provides more reliable high-level information, such as identity, and is partially aligned to input, helping to compensate for the low-level details in regions where $y_{c}$ has significant degradation. Based on this observation, our approach efficiently and comprehensively leverages both images, enabling the effective integration of detailed and contextual information from both $y_{c}$ and $y_{r}$.

In this situation, simply summing the gradients of the losses at each level can lead to conflicting gradients. To address this issue, we establish a proper pivot gradient for the loss at each feature level and align other gradients to this pivot when conflicts arise. This approach allows us to fully harness the distinct advantages of both $y_{c}$ and $y_{r}$. Specifically, for the loss using low-level features, the gradient from the loss using $y_{c}$ is prioritized, and the gradient of the loss using $y_{r}$ is modified by projecting it onto the plane orthogonal to $y_{c}$ when a conflict arises. Conversely, for the loss using high-level features, the gradient from the loss using $y_{r}$ is emphasized, and the gradient from the loss using $y_{c}$ is projected onto that of $y_{r}$ to avoid conflict and fully utilize the information in $y_{r}$. Additionally, if the magnitude of the adjusted gradient exceeds that of the pivot gradient, it is adaptively scaled according to the ratio between the two, placing greater emphasis on the pivot. As exemplified in Figure 1, the proposed PDGrad outperforms previous methods by preserving the properties of the prioritized image at each feature level while selectively extracting elements of the properties of other images in a manner that aligns with the prioritized image.

In summary, the proposed method provides the following key contributions:

- We propose a novel gradient adjustment method called PDGrad for RefBFR within a training-free guided diffusion framework.
- The loss function of the proposed method consists of two components: low-level and high-level losses, where both the coarsely restored image and the reference image are fully incorporated.
- Our proposed PDGrad establishes a proper pivot gradient for the loss at each level and adjusts other gradients to align with this pivot by modifying their direction and magnitude, thereby mitigating gradient interference.
- Extensive comparisons show the superiority of our method against previous state-of-the-art RefBFR methods.

In this paper, we outline the organization as follows: Section 2 discusses previous works on blind face restoration. Section 3 provides detailed explanation of the proposed PDGrad. In Section 4, we compare and analyze the experimental outcomes of several methods, including our proposed approach. Finally, in Section 5, we discuss the conclusions.

## 2. Related Works

Most recent BFR studies have focused on utilizing face-specific prior information, such as geometric facial priors, reference priors and generative facial priors. Note that our proposed method can be viewed as a study that exploits both reference priors and generative facial priors.

- **Geometric Facial Priors.**

Unlike natural images, faces consist of a common structural shape and components (e.g., eyes, nose, mouth and hair). Inspired by this, several approaches have been proposed to utilize the geometric priors. including facial landmarks [18,19], semantic segmentation map [20,21,22,23] and 3D shapes [24,25,26]. However, as pointed out by [16,27], such priors have limitations in guiding the fine details and texture information of the face (e.g., wrinkles and eye pupils). Furthermore, estimating geometric face priors from severely degraded inputs makes it difficult to obtain reliable results, which can affect performance.

- **Reference Priors.**

Various methods [8,9,11] have been developed to utilize high-quality facial images of the same individual as reference for restoration, aiming to leverage the distinct facial features of each person. However, these methods heavily rely on reference images of the same individual, which cannot be easily accessible. To mitigate this issue, DFDNet [10] utilizes a facial component dictionary as reference information. However, their approach may be sub-optimal for face restoration tasks since their dictionary is extracted from the pre-trained face recognition model. Inspired by [10], DMDNet [12] introduces dual dictionaries that extend beyond a single general dictionary, allowing for more flexible handling of degraded inputs, regardless of whether reference images are present. While [10,12] utilize the facial component dictionary extracted from the face recognition model, ENTED [28] introduces a vector quantized dictionary along with a latent space refinement technique. In contrast to the above methods that leverage a single reference image, ASFFNet [11] utilizes multiple reference images to select the most suitable guidance image and learns the landmark weights to improve the reconstruction quality. ENTED [28] is a blind face restoration framework that uses a high-quality reference image to restore a single degraded input image. It substitutes corrupted semantic features with high-quality codes, inspired by vector quantization, and generates style codes containing high-quality texture information. PFStorer [29] utilizes a diffusion model for face restoration and Super-Resolution, using several images of the individual’s face to customize the restoration process while preserving fine details.

- **Generative Facial Priors.**

Recently, numerous studies have widely leveraged the capability of generative models such as the Generative Adversarial Network (GAN) [30], Vector Quantized-Variational AutoEncoder (VQVAE) [31], Vector Quantized-Generative Adversarial Network (VQGAN) [32] and Denoising diffusion probabilistic models (DDPMs) [33,34,35]. GAN inversion-based methods [36,37] try to find the closest latent vector in the GAN latent space corresponding to a given input image. GFP-GAN [27] and GPEN [38] design their encoder networks to effectively find the latent vector for an input image, then utilize the pre-trained GAN model as a decoder in their methods. VQFR [39] uses a vector-quantized (VQ) codebook as a dictionary to enhance high-quality facial details. By employing a parallel decoder for the fusion of input features with texture features from the VQ codebook, this approach preserves fidelity while achieving detailed facial restoration. CodeFormer [16] is a transformer-based architecture for code prediction that captures the global structure of low-quality facial images, enabling the generation of natural faces even from severely degraded inputs. To adapt to varying levels of degradation, a controllable feature transformation (CFT) module is included, offering a versatile balance between fidelity and quality. RestoreFormer++ [40] enhances facial image restoration by utilizing fully spatial and multi-head cross-attention to merge contextual, semantic and structural information from degraded face features with high-quality priors. PMRF [41] presents an algorithm that predicts the posterior mean and then uses a rectified flow model to transport it to a high-quality image.

Building on the powerful generative capabilities of the diffusion model [33,34,35], several studies [42,43,44,45] have explored its application for BFR, and several additional studies [13,44,46,47] have also explored its application for BFR. DR2 [46] proposes a two-stage framework DR2E for blind face restoration that uses a pretrained diffusion model to remove various types of degradation and a module for detail enhancement and upsampling, and, furthermore, eliminates the need for synthetically degraded data during training. IPC [47] proposes a conditional diffusion-based BFR framework like SR3 to restore severely degraded face images. This framework employs a region-adaptive strategy that enhances restoration quality while preserving identity information. DifFace [44] establishes a posterior distribution for mapping LQ images to HQ counterparts via a pre-trained diffusion model. To achieve this, the approach estimates a transition distribution from the LQ input image to an intermediate noisy image using a diffuse estimator within the diffusion model to enhance robustness to severe degradations. Additionally, it incorporates a Markov chain that transitions the intermediate image to the HQ target image by repeatedly applying a pre-trained diffusion model, which further improves face restoration performance. Lu et al. [48] propose a diffusion-based architecture that incorporates 3D facial priors. These priors are derived from a reconstructed 3D face from an initially restored image and are integrated into the diffusion reverse process to provide structural and identity information.

However, these studies have not been developed to effectively leverage reference images for further enhancement. Meanwhile, PGDiff [13] proposed a partial guidance approach that is extensible to utilizing a reference image. By incorporating identity loss into the diffusion-based restoration method, it outperforms existing diffusion-prior-based methods. Inspired by this, we also propose a method that incorporates both diffusion prior and reference prior. However, unlike PGDiff [13], which does not consider the conflicts between gradients arising from multiple losses, our approach effectively addresses these conflicts through the integration of the proposed PDGrad, leading to more consistent and high-quality results for RefBFR.

## 3. Proposed Method

In this section, we provide a preliminary overview of the guided diffusion models to aid understanding of our proposed method in Section 3.1. We then detail the overall process of the proposed method in Section 3.2. Section 3.3 describes the proposed loss function, designed to fully leverage both the coarsely restored image and the reference image at each feature level. Lastly, in Section 3.4, our proposed PDGrad is explained, which is developed to mitigate the conflicting gradient problem.

### 3.1. Preliminary

#### 3.1.1. Denoising Diffusion Probabilistic Models

Recently, diffusion models [33,34,35] are one of the probabilistic generative models that have achieved remarkable success in the field of image generation. The diffusion model consists of a forward process and a reverse process. The forward process gradually adds Gaussian noise to an input image, while the reverse process removes the noise and reconstructs the image from the noisy state.

For an unconditional diffusion model [33] with discrete steps *T*, there exists a transition distribution $q(x_{t+1}|x_{t})$ at each step t∈{1,2,3,···,T} with corresponding variance schedule $\beta_{t}$:

(1) $$q\left(x_{t}|x_{t-1}\right)=N\left(x_{t};\sqrt{1-\beta_{t}}x_{t-1},\beta_{t}I\right),$$

where $x_{t-1}$ and $x_{t}$ are samples at time t−1 and *t*, respectively. $x_{t}$ is sampled using the reparameterization trick. $x_{t}$ can be sampled directly from $x_{0}$:

(2) $$x_{t}=\sqrt{{\bar{\alpha }}_{t}}x_{0}+\sqrt{1-{\bar{\alpha }}_{t}}\epsilon ,$$

where ${\bar{\alpha }}_{t}=\prod_{i=1}^{t}\alpha_{i}$, $\alpha_{t}=1-\beta_{t}$ and ϵ∼N(ϵ;0,I). The sampling process begins with a pure Gaussian noise $x_{T}∼N\left(x_{T};0,I\right)$ and gradually conducts the denoising step. Practically, the ideal denoising step is approximated by $p_{\theta }\left(x_{t-1}|x_{t}\right)$ [15] as follows.

(3) $$p_{\theta }\left(x_{t-1}|x_{t}\right)=N\left(\mu_{\theta }\left(x_{t},t\right),\Sigma_{\theta }\left(x_{t},t\right)\right),$$

where $\mu_{\theta }\left(x_{t},t\right)$ represents the mean, which is obtained as a linear combination of $x_{t}$ and an estimated noise $\epsilon_{\theta }\left(x_{t},t\right)$, while $\Sigma_{\theta }\left(x_{t},t\right)$ denotes the variance, a constant dependent on the pre-defined $\beta_{t}$. From Equation (2), ${\hat{x}}_{0|t}$ can be directly computed from $\epsilon_{\theta }$ as:

(4) $${\hat{x}}_{0|t}=\frac{1}{\sqrt{{\bar{\alpha }}_{t}}}x_{t}-\sqrt{\frac{1-{\bar{\alpha }}_{t}}{{\bar{\alpha }}_{t}}}\epsilon_{\theta }\left(x_{t},t\right).$$

ADM [15] introduces guided diffusion to control the sample generation of the diffusion model by leveraging an external classifier $p_{\phi }\left(c|x\right)$ that predicts the conditioning information *c* such as class label. By utilizing the classifier, the conditional distribution for denoising step in Equation (3) is approximated as a Gaussian distribution and formulated as:

(5) $$p_{\theta ,\phi }\left(x_{t-1}|x_{t},c\right)\approx N\left(\mu_{\theta }\left(x_{t},t\right)+s\Sigma_{\theta }\left(x_{t},t\right)g,\Sigma_{\theta }\left(x_{t},t\right)\right),$$

where *s* denotes the strength of classifier guidance for control. Here, the diffusion of the unconditional sampling distribution is guided by the gradient *g* towards conditional target *c*, which can be written as:

(6) $$g=\nabla_{x}\log p_{\phi }\left(c|x\right){|}_{x=\mu_{\theta }\left(x_{t},t\right)}.$$

#### 3.1.2. Partial Guidance

Recently, PGDiff [13] has introduced a training-free method and utilized classifier guidance on an unconditional diffusion model for face restoration by leveraging a pre-trained network through a technique called partial guidance. Specifically, PGDiff [13] decomposes a high-quality face image into smooth semantics and high-frequency details. The smooth semantics of the face are provided by the pre-trained face restoration model, such as CodeFormer [16]. For the high-frequency details, PGDiff relies on the diffusion prior. In addition, by leveraging a reference image and incorporating identity loss into the partial guidance, PGDiff enhances the preservation of personal identity. This identity information is guided using a pre-trained face recognition network, such as ArcFace [1].

### 3.2. Overview of Our Method

**Figure 2:**
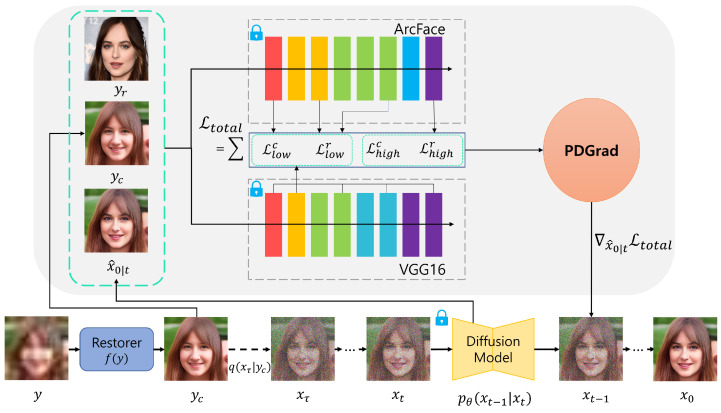
Overview of the proposed method. During the sampling process, the gradients are carefully adjusted by our PDGrad technique to prevent conflicts between gradients. This ensures that the diffusion process is efficiently guided, optimizing the quality and stability of the generated images.

Figure 2 illustrates an overview of the proposed process. Let $y\in R^{H\times W\times C}$ be the given LQ image and $y_{r}\in R^{H\times W\times C}$ be the reference HQ image. Our goal is to predict a HQ image $x_{0}\in R^{H\times W\times C}$ by adjusting conflicting gradient of the loss within a guided diffusion framework [44].

To this end, following PGDiff [13], a coarsely restored image $y_{c}\in R^{H\times W\times C}$ is first obtained by adopting a pre-trained face restoration model f(·), which can be written as:

(7) $$y_{c}=f\left(y\right).$$

However, unlike PGDiff [13], which begins the reverse process from pure Gaussian noise, our method starts from $x_{\tau }$, sampled from $y_{c}$ to enhance initialization and decrease the number of sampling steps [49]. As in Equation (2), $x_{\tau }$ can be defined as:

(8) $$x_{\tau }=\sqrt{{\bar{\alpha }}_{\tau }}y_{c}+\sqrt{1-{\bar{\alpha }}_{\tau }}\epsilon ,$$

where τ∈[0,T] is a hyperparameter that determines the starting reverse process. Also, ${\bar{\alpha }}_{\tau }=\prod_{i=1}^{\tau }\alpha_{i}$ and ϵ∼N(ϵ;0,I). Then, the reverse diffusion process is iteratively performed using the guided diffusion model [33] as follows:

(9) $$x_{t-1}∼N\left(\mu_{\theta }\left(x_{t},t\right)-s\Sigma_{\theta }\left(x_{t},t\right)\nabla_{{\hat{x}}_{0|t}}L_{total},\Sigma_{\theta }\left(x_{t},t\right)\right),$$

where *s* represents the guidance strength and $\Sigma_{\theta }\left(x_{t},t\right)$ is the time-dependent constant, as defined in Equation (5). ${\hat{x}}_{0|t}$ is the predicted image at timestep *t* (Equation (4)). $\nabla_{{\hat{x}}_{0|t}}L_{total}$ denotes the gradient of the total loss with respect to ${\hat{x}}_{0|t}$. Details of $L_{total}$ and the computation of the corresponding gradient $\nabla_{{\hat{x}}_{0|t}}L_{total}$ are discussed in the following.

### 3.3. Loss Function

One key goal of our method is to decompose the gradients from both the coarsely restored image and the reference image into their respective low-level and high-level components and then use these as guidance. This enables our approach to effectively leverage both low-level and high-level features from the coarsely restored image and the reference image.

Our total loss $L_{total}$ in Equation (9) at arbitrary diffusion time *t* is formulated by:

(10) $$L_{total}=L_{low}+L_{high},$$

where $L_{low}$ and $L_{high}$ represent losses for low-level and high-level features, respectively. For ease of notation, we omit the denoising timestep *t*. The former focuses on preserving low-level information such as face shape, edges and color, while the latter focuses on promoting high-level information such as face identity.

The proposed loss is computed using the coarsely restored image $y_{c}$, the reference image $y_{r}$, and the predicted image ${\hat{x}}_{0|t}$ (Equation (4)) at an arbitrary diffusion time *t*. To explicitly incorporate face information from $y_{r}$ and $y_{c}$ into the diffusion process, various levels of intermediate features are extracted from pre-trained ArcFace [1] and VGG16 [50] networks. As discussed in [51], it is well established that the intermediate output feature maps of the early layers of a well-trained network capture the low-level information of the input image, while the later layers capture higher-level features. Motivated by this, various levels of features are extracted from pre-trained convolutional networks such as ArcFace [1] and VGG16 [50].

Specifically, let ${\left\{u^{i}\left(z\right)\right\}}_{i=1}^{4}$ represent a set of features extracted using ArcFace [1], which is the face recognition network to determine whether two images are of the same identity. Here, $u^{i}\left(z\right)$ denotes the feature extracted from the i−th intermediate layer of the ArcFace network for the input image $z\in R^{H\times W\times C}$. In the case of low-level loss, we additionally use the VGG16 [50] network trained on a dataset that reflects human perceptual similarity to better match human preferences. Let ${\left\{v^{i}\left(z\right)\right\}}_{i=1}^{5}$ represent a set of features extracted using the VGG16 [50], where $v^{i}\left(z\right)$ denotes the feature extracted from the i−th intermediate layer of the VGG16 [50] for the input image $z\in R^{H\times W\times C}$. The specific layers used in *u* and *v* are explained in the implementation details in Section 4.1. Now, we explain each loss in detail in the following subsection.

#### 3.3.1. Low-Level Loss

The proposed low-level loss $L_{low}$ is defined as sum of two losses:

(11) $$L_{low}=L_{low}^{c}+L_{low}^{r},$$

where $L_{low}^{c}$ represents the loss that measures the similarity between the low-level features of ${\hat{x}}_{0|t}$ and $y_{c}$. Similarly, $L_{low}^{r}$ represents the loss that ensures alignment of the low-level features between ${\hat{x}}_{0|t}$ and $y_{r}$. $L_{low}^{c}$ and $L_{low}^{r}$ are defined by using pre-trained networks, including ArcFace [1] and VGG16 [50]. Concretely, $L_{low}^{c}$ is defined as:

(12) $$L_{low}^{c}=\sum_{i=1}^{3}d_{arc}\left(u^{i}\left({\hat{x}}_{0|t}\right),u^{i}\left(y_{c}\right)\right)+\sum_{i=1}^{5}d_{vgg}\left(v^{i}\left({\hat{x}}_{0|t}\right),v^{i}\left(y_{c}\right)\right).$$

Here, $d_{arc}\left(\cdot ,\cdot \right)$ [1] is defined as:

(13) $$d_{arc}\left(j_{1},j_{2}\right)=1-\frac{j_{1}\cdot j_{2}}{∥j_{1}∥∥j_{2}∥},$$

where $j_{1}$ and $j_{2}$ represent input vectors to measure distance. The distance function $d_{vgg}\left(\cdot ,\cdot \right)$ [50] is defined as:

(14) $$d_{vgg}\left(z_{1},z_{2}\right)=\sum_{l}\frac{1}{H_{l}W_{l}}\sum_{h,w}\left|\right|{w^{l}}_{hw}⊙\left({z_{1}}_{hw}^{l}-{z_{2}}_{hw}^{l}\right){\left|\right|}_{2}^{2},$$

where $z_{1}$ and $z_{2}$ are input images being compared, and ${z_{1}}_{hw}^{l}$ and ${z_{2}}_{hw}^{l}$ represent the feature values of the feature maps in the l−th layer at the spatial location (h,w) for $z_{1}$ and $z_{2}$, respectively. $H_{l}$ and $W_{l}$ denote the height and width of the feature map at the l−th layer, and ${w^{l}}_{hw}$ is a weighting factor for the feature map difference at spatial location (h,w). Symbol ⊙ represents element-wise multiplication.

Similarly, to enforce the alignment of low-level features between between $x_{0|t}$ and $y_{r}$, $L_{low}^{r}$ is formulated as:

(15) $$L_{low}^{r}=\sum_{i=1}^{3}d_{arc}\left(u^{i}\left({\hat{x}}_{0|t}\right),u^{i}\left(y_{r}\right)\right)+\sum_{i=1}^{5}d_{vgg}\left(v^{i}\left({\hat{x}}_{0|t}\right),v^{i}\left(y_{r}\right)\right).$$

#### 3.3.2. High-Level Loss

The proposed high-level loss $L_{high}$ is designed to measure the identity similarity between $x_{0|t}$ and $y_{r}$ as well as between $x_{0|t}$ and $y_{c}$. Accordingly, $L_{high}$ is comprised of two loss terms as:

(16) $$L_{high}=L_{high}^{c}+L_{high}^{r},$$

where $L_{high}^{c}$ and $L_{high}^{r}$ are defined as follows.

(17) $$L_{high}^{c}=d_{arc}\left(u^{4}\left({\hat{x}}_{0|t}\right),u^{4}\left(y_{c}\right)\right).$$

(18) $$L_{high}^{r}=d_{arc}\left(u^{4}\left({\hat{x}}_{0|t}\right),u^{4}\left(y_{r}\right)\right).$$

Here, $d_{arc}\left(\cdot ,\cdot \right)$ refers to the cosine distance metric defined in Equation (13).

### 3.4. The Proposed PDGrad

Inspired by the PCGrad [17], we propose a gradient adjustment method for guiding diffusion models in RefBFR. Similar to PGDiff [13], the unconditioned diffusion model is guided using classifier guidance. In this context, the gradient of each loss in Equation (10) acts as a specific guidance, defined by the following equation:

(19) $$g_{total}=g_{low}+g_{high},$$

where $g_{total}$, $g_{low}$, and $g_{high}$ denote the gradients $\nabla_{{\hat{x}}_{0|t}}L_{total}$, $\nabla_{{\hat{x}}_{0|t}}L_{low}$, and $\nabla_{{\hat{x}}_{0|t}}L_{high}$, respectively. Unlike PCGrad [17], we select a pivot gradient for each loss and adjust the other gradient by projecting it to the normal plane of the pivot gradient when a conflicting gradient occurs. This prevents the interfering component from being applied in the pivot direction.

From Equation (11), $g_{low}$ consists of $g_{low}^{c}=\nabla_{{\hat{x}}_{0|t}}L_{low}^{c}$ and $g_{low}^{r}=\nabla_{{\hat{x}}_{0|t}}L_{low}^{r}$, where the former is defined using $y_{c}$, and the latter is defined using $y_{r}$. When the angle between $g_{low}^{c}$ and $g_{low}^{r}$ is larger than $90^{\circ }$, that is, the cosine similarity between them is a negative value, it indicates that two gradients conflict [17]. In this case, the resultant gradient $g_{low}$ would be suboptimal as guidance for the guided diffusion. Thus, the proposed gradient $g_{low}^{pd}$, which replaces $g_{low}$, is defined as follows:

(20) $$\begin{array}{cc} g_{low}^{pd} & =\left\{\begin{array}{cc} g_{low}^{c}+g_{low}^{r} & \mathrm{if}\;\;g_{low}^{c}\cdot g_{low}^{r}\geq 0,\\ g_{low}^{c}+k_{l}\cdot {\hat{g}}_{low}^{r} & \mathrm{otherwise}. \end{array}\right. \end{array}$$

**Figure 3:**
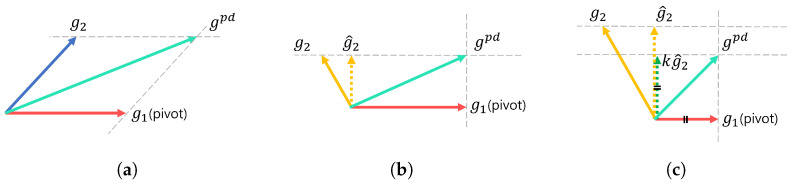
The proposed PDGrad. We illustrate an example of calculating the proposed gradient $g^{pd}$, where two input gradients are denoted as $g_{1}$ and $g_{2}$. The pivot gradient, denoted as $g_{1}$ and represented by the red arrow, is without loss of generality. In (**a**), when the gradients $g_{1}$ and $g_{2}$ do not conflict, the resultant gradient $g^{pd}$ is defined as the simple sum of the two gradients, expressed as $g^{pd}=g_{1}+g_{2}$. In (**b**), $g_{1}$ and $g_{2}$ exhibit conflicting directions. In this case, $g_{2}$ is projected onto the normal plane of the pivot gradient, resulting in ${\hat{g}}_{2}$, where the magnitude of ${\hat{g}}_{2}$ is smaller than that of $g_{1}$. Then, $g^{pd}$ is defined as $g^{pd}=g_{1}+{\hat{g}}_{2}$. In (**c**), if the magnitude of ${\hat{g}}_{2}$ is larger than that of the pivot gradient $g_{1}$, the magnitude of ${\hat{g}}_{2}$ is adjusted by scaling factor *k*, ensuring that it does not exceed that of $g_{1}$. This results in $g^{pd}=g_{1}+k\cdot {\hat{g}}_{2}$.

When two gradients $g_{low}^{c}$ and $g_{low}^{r}$ do not conflict, $g_{low}^{pd}$ is the same as the original $g_{low}$. When they conflict, we hypothesize that $y_{c}$ contains more reliable information for low-level features. Thus, as shown in Figure 3, we set the pivot direction as $g_{low}^{c}$ and define ${\hat{g}}_{low}^{r}$ by projecting $g_{low}^{r}$ onto the normal plane of $g_{low}^{c}$, which is formulated as:

(21) $${\hat{g}}_{low}^{r}=g_{low}^{r}-\frac{g_{low}^{r}\cdot g_{low}^{c}}{∥g_{low}^{c}{∥}^{2}}g_{low}^{c}.$$

The weighting factor $k_{l}$ in Equation (20) is defined by

(22) $$\begin{array}{cc} k_{l} & =\left\{\begin{array}{cc} 1 & \mathrm{if}\;\;∥g_{low}^{c}∥\geq ∥{\hat{g}}_{low}^{r}∥,\\ \frac{∥g_{low}^{c}∥}{∥{\hat{g}}_{low}^{r}∥} & \mathrm{otherwise}. \end{array}\right. \end{array}$$

Note that when the norm of the projected gradient ${\hat{g}}_{low}^{r}$ is larger than that of $g_{low}^{c}$, we clip the norm of ${\hat{g}}_{low}^{r}$ by controlling the value of $k_{l}$. This weighting factor helps our model to focus on the low-level features of $y_{c}$.

Similarly, $g_{high}$ consists of $g_{high}^{c}=\nabla_{{\hat{x}}_{0|t}}L_{high}^{c}$ and $g_{high}^{r}=\nabla_{{\hat{x}}_{0|t}}L_{high}^{r}$. For the high-level features, $y_{r}$ contains more suitable information than that of $y_{c}$. In this case, we define the modified gradient $g_{high}^{pd}$ which replaces $g_{high}$ as:

(23) $$\begin{array}{cc} g_{high}^{pd} & =\left\{\begin{array}{cc} g_{high}^{c}+g_{high}^{r} & \mathrm{if}\;\;g_{high}^{c}\cdot g_{high}^{r}\geq 0,\\ k_{h}\cdot {\hat{g}}_{high}^{c}+g_{high}^{r} & \mathrm{otherwise}. \end{array}\right. \end{array}$$

As shown in Figure 3, we set the pivot direction for $g_{high}$ as $g_{high}^{r}$ and define ${\hat{g}}_{high}^{c}$ by projecting $g_{high}^{c}$ onto the normal plane of $g_{high}^{r}$, which is formulated as:

(24) $${\hat{g}}_{high}^{c}=g_{high}^{c}-\frac{g_{high}^{c}\cdot g_{high}^{r}}{∥g_{high}^{r}{∥}^{2}}g_{high}^{r}.$$

The weighting factor $k_{h}$ in Equation (23) is defined by

(25) $$\begin{array}{cc} k_{h} & =\left\{\begin{array}{cc} 1 & \mathrm{if}\;\;∥g_{high}^{r}∥\geq ∥{\hat{g}}_{high}^{c}∥,\\ \frac{∥g_{high}^{r}∥}{∥{\hat{g}}_{high}^{c}∥} & \mathrm{otherwise}. \end{array}\right. \end{array}$$

When the norm of the projected gradient ${\hat{g}}_{high}^{c}$ is larger than that of $g_{high}^{r}$, we clip the norm of ${\hat{g}}_{high}^{c}$ by controlling the value of $k_{h}$.

Finally, the total gradient $g_{total}^{pd}$ for guiding the diffusion model can be obtained by summing $g_{low}^{pd}$ in Equation (20) and $g_{high}^{pd}$ in Equation (23). It is formulated as follows:

(26) $$g_{total}^{pd}=g_{low}^{pd}+g_{high}^{pd}.$$

The overall pipeline of the proposed PDGrad is described in Algorithm 1.
**Algorithm 1** Restoration process of PDGrad1:**Input:** a low-quality image *y*, reference image $y_{r}$, a diffusion model ($\mu_{\theta }\left(x_{t},t\right),\Sigma_{\theta }\left(x_{t},t\right)$), face restorer f(·), gradient scale *s* and the initial timestep τ2:**Output:** restored image $x_{0}$3:$y_{c}\leftarrow f\left(y\right)$4:Sample $x_{\tau }$ from $q(x_{\tau }|y_{c})$ according to Equation (8)5:**for** t **do** from τ to 16:    $\mu ,\Sigma \leftarrow \mu_{\theta }\left(x_{t},t\right),\Sigma_{\theta }\left(x_{t},t\right)$7:    ${\hat{x}}_{0|t}\leftarrow \frac{1}{\sqrt{{\bar{\alpha }}_{t}}}x_{t}-\sqrt{\frac{1-{\bar{\alpha }}_{t}}{{\bar{\alpha }}_{t}}}\epsilon_{\theta }\left(x_{t},t\right)$8:    Compute gradients $g_{low}^{pd}$ according to Equation (20)9:    Compute gradients $g_{high}^{pd}$ according to Equation (23)10:    $g_{total}^{pd}\leftarrow g_{low}^{pd}+g_{high}^{pd}$11:    $x_{t-1}\leftarrow \mathrm{sample}\;\mathrm{from}\;N\left(\mu -s\Sigma g_{total}^{pd},\Sigma \right)$12:**end for**

## 4. Experiments

As mentioned in Li et al. [10], the RefBFR methods generally outperform single-image BFR methods, since reference image contains rich textures and the fine detains lost in the given LQ image [52,53]. Hence, in this paper, we mainly compare our proposed method with the recent reference-based BFR methods such as ASFFNet [11], DMDNet [12], and PGDiff [13]. Additionally, we report comparison with the single-image BFR methods, including VQFR [39], CodeFormer [16], RestoreFormer++ [40] and DifFace [44]. All experiments in this paper are conducted using the official models with pre-trained weights provided by the authors.

In this section, we provide the details of experimental settings in Section 4.1. In Section 4.2, we compare our proposed method with the state-of-the-art BFR methods through both qualitative and quantitative analyses. Section 4.4 presents the evaluation results of the ablation study to assess the effect of each component of our proposed approach.

### 4.1. Experimental Setting

#### 4.1.1. Implementation Details

Following PGDiff [13], we utilize the pre-trained diffusion model provided by Yue et al. [44] for a fair comparison. This model is an unconditional diffusion network trained on the FFHQ dataset [54] and supports an image resolution of 512×512. In our RefBFR process, we leverage CodeFormer [16] as a pre-trained face restoration model to obtain coarsely restored image from a given LQ image, which is denoted as f(·) in Equation (7). It is noteworthy that the proposed method employs off-the-shelf pre-trained networks which are readily accessible online, without the need for additional training. The proposed framework is implemented using Pytorch [55] and the inference process is executed on a single NVIDIA GeForce RTX 3090 GPU. Empirically, we set τ for initial guidance step to 700, and gradient scale *s* to 0.1. The intermediate features ${\left\{u^{i}\left(z\right)\right\}}_{i=1}^{3}$ are extracted from layers conv1_1, conv2_2, and conv3_2 of ArcFace [1]. $u^{4}\left(z\right)$ is final output feature of ArcFace [1]. The intermediate features ${\left\{v^{i}\left(z\right)\right\}}_{i=1}^{5}$ are extracted from layers conv1_2, conv2_2, conv3_3, conv4_3 and conv5_2 of VGG16 [50].

#### 4.1.2. Datasets

To evaluate our method, we use CelebRef-HQ dataset [12], which comprises a total of 10,555 HQ face images. This dataset includes 1005 distinct identities and each individual has between 2 and 21 images. Specifically, for our evaluation, we randomly select two images from each of the 1005 identities in the dataset. Then, one image is designated as the ground-truth HQ image, while the other image serves as the reference HQ image. Following the degradation model specified in recent BFR studies [16,27,39,56], the LQ images are synthesized as follows:

(27) $$y={\left[{\left[{\left(x_{GT}⊗k_{\sigma }\right)}_{↓r}+n_{\delta }\right]}_{JPEG}\right]}_{↑r},$$

where the ground-truth HQ image $x_{GT}$ is first blurred using a Gaussian kernel $k_{\sigma }$, then downsampled by a scale factor *r*. Next, Gaussian noise $n_{\delta }$ is added, followed by JPEG compression with quality factor *q* is applied. Lastly, the LQ image *y* is resized back to 512×512. In this paper, we randomly sample σ, *r*, δ, and *q* from [0.1,15],[24,40],[0,20], and [30,100], respectively.

#### 4.1.3. Evaluation Metrics

For a quantitative evaluation, we employ PSNR, SSIM [57] and NIQE [58], which are commonly used metrics in the image restoration field. Additionally, we measure LPIPS [50] to assess perceptual similarity between ground-truth images and restored images. Furthermore, FID [59] is used to quantify the distance between the feature distributions of HQ face datasets and restored images. We employ the CelebRef-HQ dataset [12] to measure the feature distributions of HQ face dataset. To measure the similarity in facial identity between the ground-truth images and the restored images, we compute the angle between their embedding vectors using ArcFace [1], denoted as Deg [39]. We also compare the landmark distance (LMD) [39], which is calculated as the average L2 distance of 98 facial landmarks predicted using Awing [60] between the ground-truth images and the restored images.

### 4.2. Quantitative Comparison

The quantitative comparisons of various RefBFR methods are shown in Table 1. Here, ASSFNet [11] and DMDNet [12] are face restoration methods that incorporate landmark estimation procedures. However, for certain LQ input images, these methods struggle with accurate landmark detection, preventing them from generating results and rendering testing infeasible. Therefore, to ensure a fair comparison between methods, we conduct experiments on 662 LQ input images, a subset of the CelebRef-HQ dataset [12], where testing with ASSFNet [11] and DMDNet [12] is feasible, as shown in Table 1. The results demonstrate that the proposed PDGrad achieves better performance than other methods in terms of LPIPS, Deg, LMD and NIQE. PDGrad achieved a |0.4508−0.4437|/0.4508=1.57% better result in terms of LPIPS compared to the second best competitive method, indicating that it consistently restores faces with perceptual quality closest to the ground truth. For fidelity, the proposed PDGrad achieved the highest performance in terms of Deg and LMD, compared to the second best method with improvements of |55.53−53.1|/55.53=4.38% and |6.25−6.01|/6.25=3.84%, respectively. This demonstrates that our method can accurately recover facial identity similarity and details. Additionally, in terms of image quality metrics such as NIQE, the proposed PDGrad outperforms DMDNet [12] by |3.85−3.38|/3.85=12.21% and produces more realistic details. This can be attributed to the incorporation of (1) the proposed loss function that considers perceptual quality and identity preservation and (2) the proposed gradient adjustment procedure that effectively handles the conflict of the gradients.

In Table 2, we further compared the proposed PDGrad with PGDiff [13] using the full CelebRef-HQ dataset [12], which consists of 1005 LQ input images. Compared to PGDiff, PDGrad shows outperforming results on the LPIPS, Deg, LMD and NIQE metrics. Notably, we achieved improvements of |56.68−53.90|/56.68=4.9% and |4.32−3.39|/4.32=21.53% in Deg and NIQE, respectively. This indicates that PDGrad generates face images that are more faithful to the ground truth identity while maintaining high image quality during the guided diffusion process.

Table 3 provides a quantitative comparison between the proposed PDGrad and single-image BFR methods on the full CelebRef-HQ dataset [12]. The results demonstrate that our method achieves better or at least comparable performance in terms of LPIPS, Deg, LMD, NIQE and FID. While CodeFormer [16] achieves a higher perceptual quality than other methods according to LPIPS, its identity similarity is significantly compromised according to Deg. Although our proposed PDGrad is slightly worse in LPIPS compared to CodeFormer [16], it excels at preserving identity similarity, as measured by Deg. Notably, our method demonstrates a significant improvement |68.3−53.9|/68.3=21.08% in Deg compared to the second-best model.

### 4.3. Qualitative Comparison

**Figure 4:**
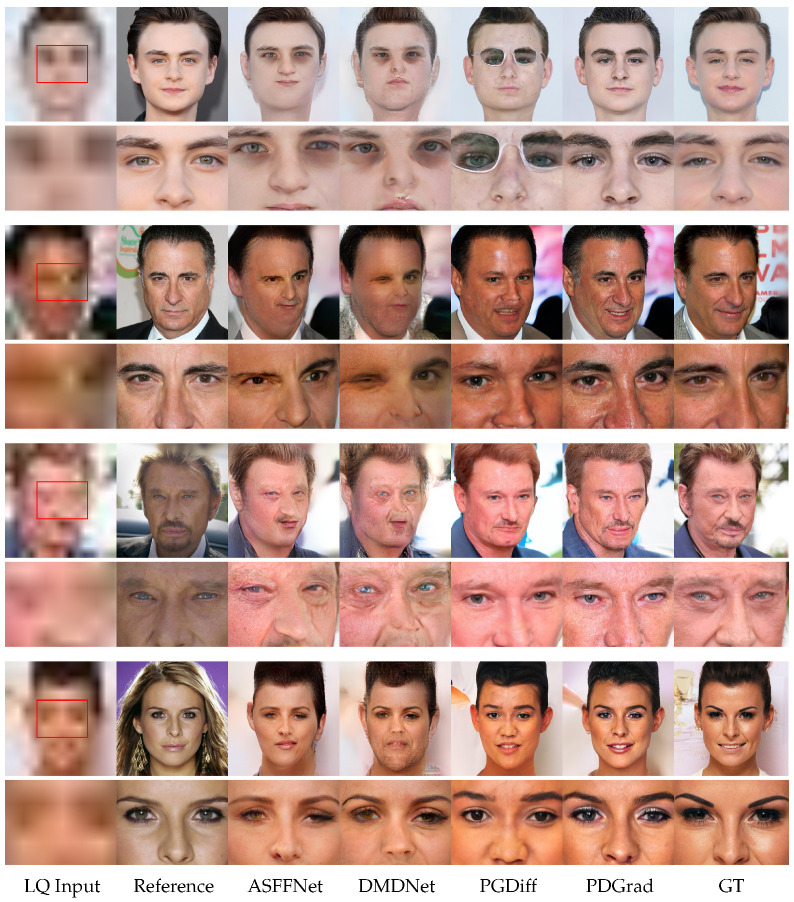
Qualitative comparison of RefBFR methods on the CelebRef-HQ dataset [12]. For a better comparison of visual quality, zooming-in is recommended. From left to right, LQ input image, reference image, ASFFNet [11], DMDNet [12], PGDiff [13], the proposed PDGrad and ground truth (GT).

**Figure 5:**
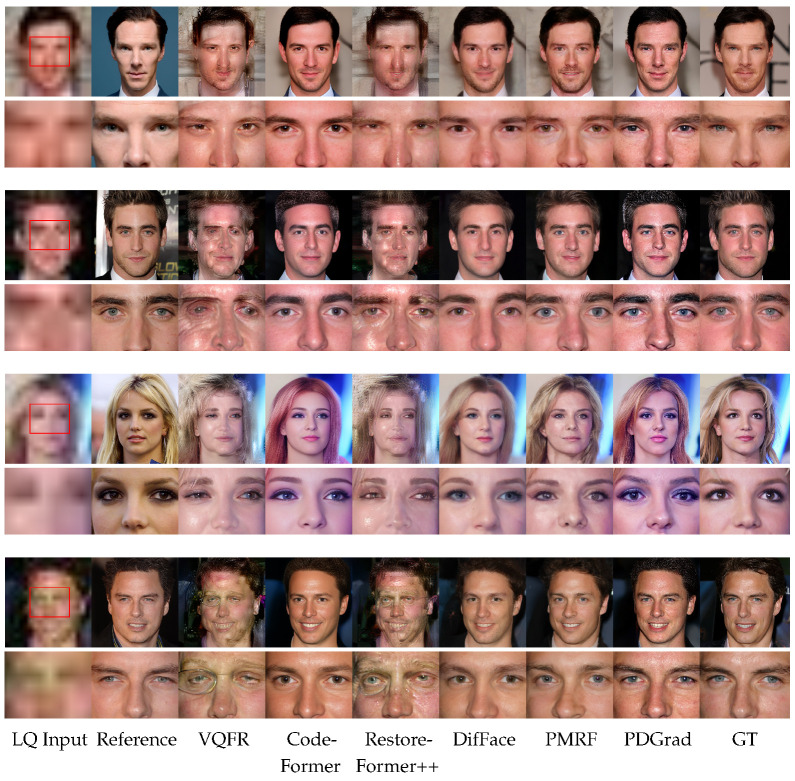
Qualitative comparison of single-image BFR methods on the CelebRef-HQ dataset [12]. For a better comparison of visual quality, zooming-in is recommended. From left to right, LQ input image, reference image, VQFR [39], CodeFormer [16], RestoreFormer++ [40], DifFace [44], PMRF [41], the proposed PDGrad and ground truth (GT).

Visual comparisons of RefBFR and single-image BFR methods are presented in Figure 4 and Figure 5, respectively. In each figure, the even-numbered rows provide close-up views that highlight specific details in the same areas indicated by red rectangles in the LQ input of the corresponding images in the odd-numbered rows. Figure 4 demonstrates that ASFFNet [11] and DMDNet [12] fail to preserve the identity and to produce a proper facial shape. Specifically, it can be observed that components like the eyes have been mostly restored, but there are difficulties in restoring most components such as the nose and mouth. PGDiff [13] is able to produce high-quality images, but it showed a lack of facial details in terms of preserving identity. Unlike other methods, our PDGrad is able to generate high-quality images with high fidelity in skin texture, wrinkles and eye shape.

In Figure 5, VQFR [39] and RestoreFormer++ [40] fail to produce satisfactory restoration results due to severe degradations. The results contain artifacts and lack facial details. CodeFormer [16], DifFace [44] and PMRF [41] produce high-quality images, but they also lack of facial details important for preserving identity. However, the proposed PDGrad exhibits superior performance over all other methods in restoring sharp and fine details of the face (e.g., in the eyes, nose and mouth). Moreover, the proposed method can generate identity-preserving results consistent with the GT while also improving the perceptual quality of the image.

### 4.4. Ablation Study

We conducted ablation studies to investigate the impact of each component in the proposed PDGrad. First, Table 4 presents the effects of the gradient adjustment components in the PDGrad, summarizing the configurations and results for each experiment. All the methods in Table 4 use the same network architectures and a loss function defined as the sum of Equations (11) and (16), with the only difference being the gradient used to guide the diffusion model. A1 in Table 4 represents a baseline model, where the total gradient in Equation (19) is obtained by simply summing of multiple gradients without any gradient adjustment. The model is then guided by this gradient during the diffusion sampling process. A2 is a method that resolves gradient conflicts by adjusting only the gradient direction. This is achieved by projecting the gradient to the normal plane of the pivot gradient when conflicts occur. In A2, the values of both $k_{l}$ and $k_{h}$ are fixed as 1 in Equations (20) and (23) for all cases, respectively. Compared to A1, A2 shows the improvements of |0.4513−0.4499|/0.4513=0.31% in LPIPS, |59.24−53.90|/59.24=9.01% in Deg, |6.49−6.36|/6.49=2% in LMD and |3.44−3.39|/3.44=1.45% in NIQE, respectively. These improvements highlight that adjustment of the gradient direction in PDGrad effectively mitigates the conflicts between gradients arising from multiple losses. Consequently, the diffusion process is more efficiently guided, enhancing the quality of the generated images. PDGrad is our proposed method, which is built upon A2 by additionally applying an adaptive scaling for $k_{h}$ and $k_{l}$ to ensure that the magnitude of the projected gradient does not exceed that of the pivot gradient. This adaptive scaling is designed to enhance the influence of the pivot gradient when applying gradient adjustment. As a result, PDGrad not only adjusts the gradient direction towards the pivot, but also preserves the influence of the pivot gradient by adjusting the magnitude of other gradients. This leads to the restoration of images with both perceptually improved and enhanced fidelity. Consequently, compared to A2, PDGrad shows further improvements of |0.4506−0.4499|/0.4506=0.16% in LPIPS and |54.02−53.90|/54.02=0.22% in Deg.

To effectively guide detailed facial information, PDGrad defines the low-level loss by combining two components, $d_{arc}$ and $d_{vgg}$, as shown in Equations (12) and (15). As shown in Table 5, to evaluate the impact of each component in these equations, we performed an additional ablation study by using either $d_{arc}$ or $d_{vgg}$ in Equations (12) and (15). The experiment was performed by varying only the low-level loss component, while applying gradient adjustment, including gradient projection and adaptive scaling. A3 shows results using $d_{arc}$ exclusively in both Equations (12) and (15), while A4 demonstrates results using $d_{vgg}$ exclusively in those equations. When A3 and A4 are compared to PDGrad, the results of PDGrad show an improvement in the LPIPS by |0.4616−0.4499|/0.4616=2.53% and |0.4632−0.4499|/0.4632=2.87%, respectively. Additionally, the Deg score improves by |60.60−53.90|/60.60=11.06% and |61.87−53.90|/61.87=12.88%, respectively. These improvements indicate that using both $d_{arc}$ and $d_{vgg}$ together is more effective in guiding perceptual and fidelity information than using either component alone.

As shown in Table 6, we conducted further experiments to explore the effects of the input images by using either the coarsely restored image $y_{c}$ from CodeFormer [16] or the reference image $y_{r}$ individually for gradient computation in the diffusion sampling process. This setup represents an extreme scenario where the angle between two input gradients derived from $y_{c}$ and $y_{r}$, respectively, are aligned in the same direction. This alignment occurs when $y_{c}$ and $y_{r}$ are identical, resulting in the same result as if either image were used alone. In Table 6, A5 represents the case of using only $y_{c}$, where $g_{low}^{pd}$ and $g_{high}^{pd}$ in Equations (20) and (23) are set to $g_{low}^{c}$ and $g_{high}^{c}$, respectively. Similarly, A6 represents the case of using only $y_{r}$, where $g_{low}^{pd}$ and $g_{high}^{pd}$ in Equations (20) and (23) are set to $g_{low}^{r}$ and $g_{high}^{r}$, respectively. Our results confirm that PDGrad, which utilizes both $y_{c}$ and $y_{r}$, as presented in Table 6, outperformed the other models across most metrics.

## 5. Conclusions

In this paper, we present a Pivot Direction Gradient (PDGrad), a novel gradient adjustment method designed to enhance reference-based blind face restoration within the guided diffusion framework. By focusing on the issue of conflicting gradients in multi loss-based guidance, the proposed method aligns gradients across different feature levels, ensuring both low-level and high-level facial characteristics are accurately restored. Through comprehensive experiments, we have demonstrated that the proposed method consistently outperforms existing methods, offering robust solution for reference-based blind face restoration. This advancement highlights the potential of gradient adjustment techniques for guided-diffusion models and the broader image restoration field.

## Figures and Tables

**Table 1:** Quantitative comparison of reference-based BFR methods on a subset of the CelebRef-HQ dataset [12]. The symbol ↑ in parentheses represents that the higher the value, the better. Similarly, the symbol ↓ indicates that the lower the value, the better. We highlight the best model and the second best model with the **bold** and underline, respectively.

Methods	Metrics						
	**LPIPS ↓**	**Deg ↓**	**LMD ↓**	**NIQE ↓**	**FID ↓**	**PSNR ↑**	**SSIM ↑**
ASFFNet [11]	0.4850	76.43	27.82	4.67	56.84	19.00	0.5759
DMDNet [12]	0.5174	77.92	24.23	3.85	80.31	19.12	0.5374
PGDiff [13]	0.4508	55.53	6.25	4.29	**26.58**	**19.15**	**0.5917**
PDGrad	**0.4437**	**53.10**	**6.01**	**3.38**	27.28	18.33	0.5332

**Table 2:** Quantitative comparison with PGDiff on the CelebRef-HQ dataset [12]. The symbol ↑ in parentheses represents that the higher the value, the better. Similarly, the symbol ↓ indicates that the lower the value, the better. We highlight the best model and the second best model with the **bold** and underline, respectively.

Methods	Metrics						
	LPIPS ↓	Deg ↓	LMD ↓	NIQE ↓	FID ↓	PSNR ↑	SSIM ↑
PGDiff [13]	0.4584	56.68	6.75	4.32	**26.58**	**18.99**	**0.5936**
PDGrad	**0.4499**	**53.90**	**6.36**	**3.39**	27.28	18.24	0.5368

**Table 3:** Quantitative comparison of single image BFR methods on the CelebRef-HQ dataset [12]. The symbol ↑ in parentheses represents that the higher the value, the better. Similarly, the symbol ↓ indicates that the lower the value, the better. We highlight the best model and the second best model with the **bold** and underline, respectively.

Methods	Metrics						
	**LPIPS ↓**	**Deg ↓**	**LMD ↓**	**NIQE ↓**	**FID ↓**	**PSNR ↑**	**SSIM ↑**
VQFR [39]	0.5332	79.19	13.93	3.48	99.85	18.32	0.4881
CodeFormer [16]	**0.4426**	70.07	7.81	5.02	34.57	18.90	0.5828
RestoreFormer++ [40]	0.5401	78.94	14.03	3.97	112.30	18.62	0.4870
DifFace [44]	0.4510	68.30	**6.25**	4.89	28.12	**20.93**	**0.6334**
PMRF [41]	0.4552	69.12	6.79	4.35	**25.43**	20.30	0.6116
PDGrad	0.4499	**53.90**	6.36	**3.39**	27.28	18.24	0.5368

**Table 4:** Ablation study on the gradient adjustment components of the proposed PDGrad using the Celebref-HQ dataset [12]. The symbol ↑ in parentheses represents that the higher the value, the better. Similarly, the symbol ↓ indicates that the lower the value, the better. We highlight the best model and the second best model with the **bold** and underline, respectively.

Ablation	Gradient Projection	Adaptive Scaling	Metrics						
			**LPIPS ↓**	**Deg ↓**	**LMD ↓**	**NIQE ↓**	**FID ↓**	**PSNR ↑**	**SSIM ↑**
A1			0.4513	59.24	6.49	3.44	**26.70**	**18.24**	**0.5434**
A2	✓		0.4506	54.02	**6.36**	**3.36**	27.43	18.23	0.5348
PDGrad	✓	✓	**0.4499**	**53.90**	**6.36**	3.39	27.28	**18.24**	0.5368

**Table 5:** Ablation study on the components of the loss function in the proposed PDGrad using the Celebref-HQ dataset [12]. The symbol ↑ in parentheses represents that the higher the value, the better. Similarly, the symbol ↓ indicates that the lower the value, the better. We highlight the best model and the second best model with the **bold** and underline, respectively.

Ablation	$d_{\mathrm{arc}}$	$d_{\mathrm{vgg}}$	Metrics						
			**LPIPS ↓**	**Deg ↓**	**LMD ↓**	**NIQE ↓**	**FID ↓**	**PSNR ↑**	**SSIM ↑**
A3	✓		0.4616	60.60	6.53	3.76	**26.42**	**18.32**	**0.5720**
A4		✓	0.4632	61.87	6.92	**3.24**	31.19	17.59	0.5165
PDGrad	✓	✓	**0.4499**	**53.90**	**6.36**	3.39	27.28	18.24	0.5368

**Table 6:** Ablation study on the input images used in the proposed PDGrad with the Celebref-HQ dataset [12]. The symbol ↑ in parentheses represents that the higher the value, the better. Similarly, the symbol ↓ indicates that the lower the value, the better. We highlight the best model and the second best model with the **bold** and underline, respectively.

Ablation	CodeFormer ($y_{c}$)	Reference ($y_{r}$)	Metrics						
			**LPIPS ↓**	**Deg ↓**	**LMD ↓**	**NIQE ↓**	**FID ↓**	**PSNR ↑**	**SSIM ↑**
A5	✓		0.4563	70.76	6.95	3.59	**25.41**	18.10	**0.5474**
A6		✓	0.5126	56.03	7.40	**3.27**	34.15	17.41	0.4988
PDGrad	✓	✓	**0.4499**	**53.90**	**6.36**	3.39	27.28	**18.24**	0.5368

## Data Availability

Data are contained within the article.
